# Ouabain-Induced Cytoplasmic Vesicles and Their Role in Cell Volume Maintenance

**DOI:** 10.1155/2015/487256

**Published:** 2015-03-19

**Authors:** M. A. Russo, E. Morgante, A. Russo, G. D. van Rossum, M. Tafani

**Affiliations:** ^1^Laboratory of Molecular and Cellular Pathology, IRCCS San Raffaele Pisana, Via di Val Cannuta 247, 00166 Roma, Italy; ^2^Department of Experimental Medicine, Sapienza University of Rome, 00161 Rome, Italy; ^3^IRCCS Regina Elena Institute, 00144 Rome, Italy; ^4^Department of Pharmacology, Temple University School of Medicine, Philadelphia, PA 19140, USA

## Abstract

Cellular swelling is controlled by an active mechanism of cell volume regulation driven by a Na^+^/K^+^-dependent ATPase and by aquaporins which translocate water along the osmotic gradient. Na^+^/K^+^-pump may be blocked by ouabain, a digitalic derivative, by inhibition of ATP, or by drastic ion alterations of extracellular fluid. However, it has been observed that some tissues are still able to control their volume despite the presence of ouabain, suggesting the existence of other mechanisms of cell volume control. In 1977, by correlating electron microscopy observation with ion and water composition of liver slices incubated in different metabolic conditions in the presence or absence of ouabain, we observed that hepatocytes were able to control their volume extruding water and recovering ion composition in the presence of ouabain. In particular, hepatocytes were able to sequester ions and water in intracellular vesicles and then secrete them at the bile canaliculus pole. We named this “vesicular mechanism of cell volume control.” Afterward, this mechanism has been confirmed by us and other laboratories in several mammalian tissues. This review summarizes evidences regarding this mechanism, problems that are still pending, and questions that need to be answered. Finally, we shortly review the importance of cell volume control in some human pathological conditions.

## 1. Introduction: “Cloudy Swelling”

“Cloudy swelling” is an early and frequent morphological cell alteration first identified by Virchow in his “Die Cellular Pathologie” [1]. Swelling is an increase of cell volume due to accumulation of water and ions in subcellular compartments. It occurs under two rather common conditions in human pathology: (a) reduction of metabolic activity leading to a decrease of energy charge (hypoxia, ischemic hypoxia [2], or uncoupling agents) and (b) damage of cell membranes with loss of physiological ionic gradients (plasma membrane rupture or pore formation, Na^+^/K^+^-ATPase malfunction, etc.). Extreme “cloudy swelling” is irreversible, leading to cell necrosis. Therefore, a clear picture of the underlying pathogenetic mechanisms of cell swelling is important to understand many aspects of human diseases.

Living cells regulate their water content even under apparently isosmotic conditions to compensate the osmotic forces due to higher intracellular concentration of charged macromolecules [3]. Regulation of cell water is necessary to maintain (a) an appropriate physical size of cells and organelles; (b) the optimal concentrations of soluble metabolic components (ions, substrates, and soluble enzymes); (c) the functional subcellular architecture and relational distances necessary for metabolism and vectorial movements into the cell. The latter includes intracellular transport (secretion, endocytosis, axonal transport, and organelles movements) and sarcomeric contraction.

*In vitro* cell swelling is rapidly induced by reducing metabolic activity (cold incubation and inhibition of energy metabolism and ATP production), by inhibiting ion transport (i.e., ouabain, abnormal external ionic concentrations), or by damaging the membrane(s) (i.e., lipoperoxidation, high temperature, ionophores, etc.). By using different* in vitro* models, Wilson [4], Leaf [5], and Macknight and Leaf [3] demonstrated the role of active transport of ions across the plasma membrane in preventing swelling under isosmotic condition. They showed that the distribution of Na^+^ was crucial and that its physiologic concentrations were maintained by a coupled transport of Na^+^ and K^+^, operated by a Na^+^/K^+^-ATPase, a pump apparently totally inhibited by ouabain both in* in vitro* and in soluble systems.

However, in 1976 and 1977, it was strongly evident that inhibition of Na/K-transport by ouabain in a model of tissue slices does not totally abolish regulation of cell volume under isosmotic conditions. To account for the ouabain-resistant fraction of volume regulation, Russo et al., for the first time, by strictly correlating ion and water distribution with ultrastructural observations, proposed that vesicles of exocytosis, highly induced in the presence of ouabain, could be responsible for water extrusion, after swelling by cold incubation [6–9].

A previous account of this proposal has been published long time ago [8]. This review aims to summarize evidences that we have at present about cellular swelling, problems that are still pending, and questions that need an answer.

## 2. Volume Regulation in Mammalian Tissues

The property of cell volume control is a fundamental feature of all cells and, in particular, of mammalian cells. Cell volume results from the content of water and ions, osmotically active soluble molecules, and macromolecules and supramolecular structures, such as organelles, filaments, polymers, and metabolites. These components are confined in intracellular space delimited by the plasma membrane. Ions and osmotically active molecules play a crucial role in steady state conditions, determining the amount of the intracellular water, which represents the major component of cytosol and intraorganellar space [10]. Changes in cell volume, indeed, may occur through* swelling*, that is, increase in water and ion content, or* hypertrophy*, which is an increase in cell constituents, such as sarcomeric filaments (muscle hypertrophy), lipid droplets (obesity), endoplasmic reticulum (drug metabolism), mitochondria, and other cell structures, following a functional adaptation. In the case of swelling, three major components determine the final volume: the amount of intracellular water, differently distributed in the various subcellular compartments, the amount of osmotically active solutes (ions and macromolecules), and the plasma membrane properties for controlling intracellular concentrations of osmotically active solutes (ions and free amino-acids) and for excluding other extracellular osmotically active molecules.

In mammalian cells, under normal conditions, the intracellular composition and the extracellular environment are almost constant, obeying to three simple principles: (1) cell osmolarity and water content must be equal to extracellular osmolarity; (2) inorganic ion distribution (Na^+^, K^+^, and Cl^−^) on two sides of plasma membrane is controlled by active/passive transport mechanisms, satisfying the basic requirement of electroneutrality; (3) water travels through aquaporins as determined by osmotic equilibrium. The localization and the isoforms of aquaporins may influence the distribution among subcellular compartments of intracellular water and an excess of water extrusion (cell shrinkage) in the presence of extracellular hyperosmolarity [11–13].

Under isosmotic conditions, cell volume regulation in mammalian is, to a large extent, explicable by passage of water directly through aquaporins of plasma membrane in response to ionic balance maintained by a Na^+^/K^+^-ATPase transport system [10], which is apparently completely inhibited by ouabain [4, 5]. However, observations on a number of mammalian tissues suggest that this ability to regulate cell volume is at least partially resistant to oubain or to the absence of K^+^ from the medium [6, 9, 14–18]. Several hypotheses have been proposed to account for this ouabain-resistant extrusion of water: (a) a “cryptic pump” [19] inaccessible to oubain, being hidden in different subcellular sites of plasma membrane, due to impermeable submembranaceous cytoskeletal network (which regulates the secretion in many endocrine and epithelial cells); (b) a second Na^+^ pump [20] insensitive to ouabain and uncoupled from K^+^ movements. Marín et al. [21] have partially characterized a Na^+^-dependent ATPase, inhibited by furosemide, in the basolateral plasma membrane of epithelial cells in rat kidney proximal tubule. (c) Kleinzeller [22], Rorive et al. [23], and others have provided several evidences that a contractile system associated to the cell periphery (submembranaceous cytoskeleton) could be responsible for the extrusion of water and ions accumulated during a period of previous swelling, thus controlling cell volume. This mechanism appears to be typically dependent on Ca^2+^ and ATP.

The work cited above was done with no published morphological and ultrastructural control. A few years later, Garfield and Daniel [15] using uterine smooth muscle and Russo et al. using liver [6, 24] and kidney slices [18, 25] have described that the presence of ouabain is associated with the formation of cytoplasmic vesicles as well with continued extrusion of water and ions and that this could be the basis of ouabain-resistant cell volume control.

The idea that intracellular vesicles (mostly round and electron-clear at TEM) can be involved in volume and osmotic regulation was not completely new. In fact, contractile vacuoles of unicellular organisms, tonoplasts of plant cells, and large vacuoles of mammalian tissues during vacuolar degeneration (hypoxia) are all different types of vesicles with similar morphology and function in sequestering water, maintaining cell volume or turgor, and avoiding cytosolic dilution. Vesicles in excess can be expelled (exocytosis), extruding water. However, ultrastructural observations of different mammalian tissues suggest that in normal conditions vesicles play a minor role in water and ion movement. In contrast, in a number of metabolic and toxic emergencies (including ouabain treatment), cytosolic water rapidly is compartmentalized in different membrane-bound structures of endoplasmic reticulum and Golgi apparatus (vesicles), mitochondria, and other organelles. Indeed, water is extruded from cytosol, nucleoplasm, and mitochondrial matrix (transition from* swelling* →* orthodox* →* condensed forms*), accumulating in vesicles derived from ER cisternae and Golgi apparatus. We will discuss below the different morphology and origin of at least two types of vesicles: one that we call “secretory” seen in the presence of ouabain and another observed in the presence of oligomycin (or oligomycin + ouabain or low doses of amytal) for cell water compartmentation [6, 26].

## 3. The Ouabain-Resistant Na^+^/K^+^-ATPase Independent Cell Volume Regulation

The experimental model adopted for the work by Russo et al. namely, preincubation of tissue slices at 1°C, followed by recovery at 37°C in the presence of various agents, has been well characterized previously [6, 8, 27, 28]. The qualitative characteristics and quantitative balance of the ionic and water exchanges taking place with and without ouabain are shown in Table 1 and in Figure 2 and are similar to the previous results obtained in many experiments and published in previous papers. Ouabain (2 mM) completely inhibited the net reuptake of K^+^ at 37°C suggesting that this concentration effectively inhibited the Na-K ATPase. Thus, increasing ouabain to 5 mM caused no further effect, while omitting K^+^ from the medium produced effects closely similar to those of ouabain [7, 29]. Further, the unidirectional influx of ^86^Rb (an analog of K^+^) was maximally inhibited by 2 mM ouabain [30]. It was concluded that the expulsion of water, Na^+^, and Cl^−^ continuing in the presence of ouabain is driven by an energy-providing mechanism other than the Na^+^/K^+^-ATPase, clearly associated with the presence of round electron-clear vesicles.

Importantly, this mechanism is highly sensitive to the decrease of ATP and involves the entry of water and ions into cytoplasmic vesicles that expand in the presence of ouabain followed by expulsion of the vesicular contents by exocytosis into the canaliculus [6, 8, 29].

**Figure 1:**
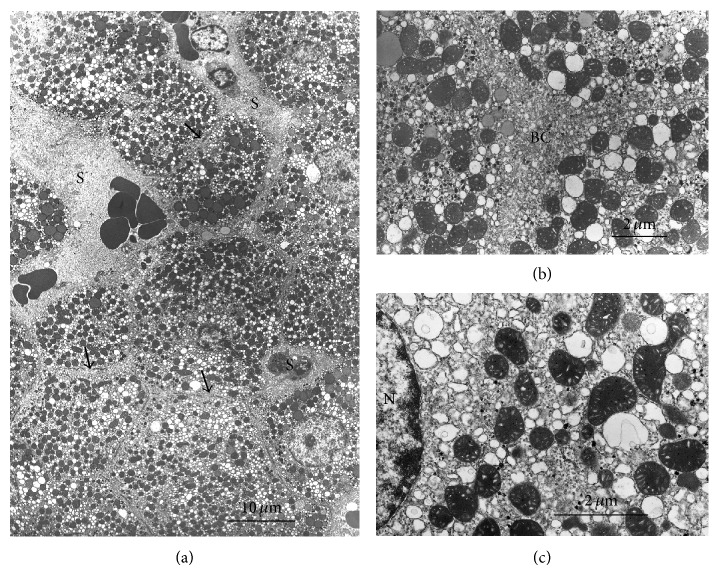
Liver slices after 90 min incubation at 1°C [6]. (a) Low magnification. Cells are highly swollen with volume increase. Cisternae of the endoplasmic reticulum are dilated; mitochondria are mostly in intermediate-condensed form, sometimes swollen. Cytosol and nucleoplasm are rather electroclear decreased and disorganized extracellular spaces. (b) Detail of dilated endoplasmic reticulum, detachment of ribosomes, and boundaries of cells that became uncertain with disorganized microvilli; increase and disorganization of extracellular spaces (sinusoidal, lateral, and canalicular regions). (c) Additional details: cisternae of the endoplasmic reticulum are dilated; mitochondria mostly in intermediate-condensed form, occasionally swollen; cytosol and nucleoplasm are rather electroclear (diluted). S = sinusoid; BC = bile canaliculus; N = nucleus.

In our model of tissue slices, cold (1°C) incubation for 90 min caused advanced swelling with accumulation of Na^+^, loss of K^+^, and retention of water distributed in the various subcellular compartments (Figures 1(a), 1(b), and 1(c)) [6].

**Figure 2:**
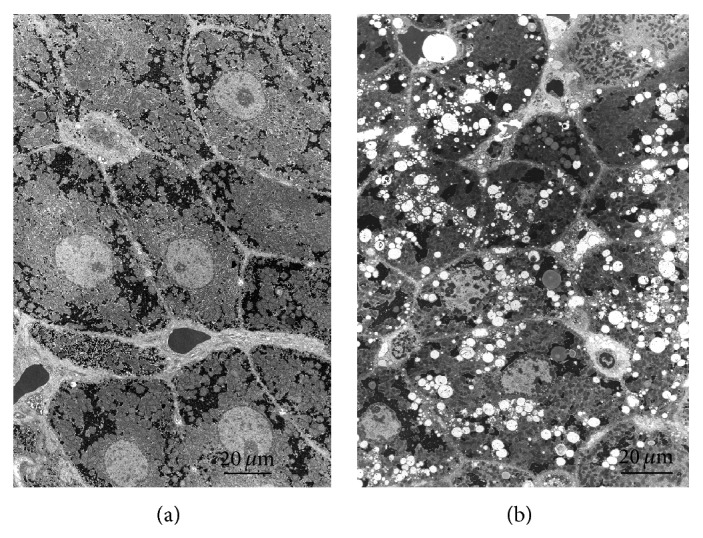
Comparison at low magnification between slices after 60 min recovery at 38°C in the presence (b) or absence (a) of 2 mM ouabain. (a) Control liver slices after incubation for 90 min at 1°C followed by 60 min at 38°C. The ultrastructure has recovered well from the alterations in the cold preincubation (Figure 1) (see [6]). The cytosol and organelles appear to have a normal electron density. (b) Liver slice after 90 min at 1°C followed by 60 min at 37°C in the presence of 2 mM ouabain. A large number of rounded, clear vesicles are present, mainly localized in the Golgi region towards and around the bile canaliculi. The ground substance and other subcellular components are similar to the control in (a).

Restoration of metabolic activity by subsequent incubation for 60 min at 38°C allows cells to extrude Na^+^, Cl^−^, and water and reaccumulate K^+^ (Table 1). Additionally, structural recovery started after 5 min of warm incubation and was complete after 60 min (Figure 2(a)).

Treatment with ouabain* in vitro* induces characteristic, rounded vesicles in tissue slices (Figure 2(b)) and/or cultured cells of uterus [16], liver [8, 25, 31], hepatoma [9], renal cortex [18], and lung [32]. In each case, the vesicles have been correlated to an ouabain-resistant mechanism of isoosmotic cell volume control. By contrast, the salt gland of salt-adapted water birds was found not to expel water in the presence of ouabain and did not show vesicles [8].

The first systematic hypothesis on the proposed mechanism and on the formation and nature of the ouabain-induced vesicles for ion and water expulsion has been summarised previously although a number of crucial questions were still pending [8].

Briefly (see also Figure 9), formation of the vesicles is dependent on the presence of Cl^−^. Vesicles appear to originate by expansion of terminal cisternae of the endoplasmic reticulum and of Golgi elements and they have an acidic content. In liver, the vesicles accumulate and secrete at the canalicular pole and in renal cortex near the basolateral infolding of the plasma membrane. In each case and most clearly in the kidney, there is evidence of their fusion with the plasma membrane suggesting expulsion of the content [18, 24].

Accordingly, it has been suggested that accumulation of water in the vesicles is driven by the Cl^−^ dependent, H^+^-transporting vacuolar adenosine triphosphatase (V-ATPase) of the vacuolar membranes [30, 33]. The vesicles are suggested to move to the cell periphery by a microfilament-dependent and microtubule-independent mechanism and then to expel their contents by exocytosis. Cytochalasins addition or absence of Ca^2+^ greatly increases the number, size, and intracellular distribution of the vesicles, suggesting an inhibition of the cytoskeletal function for their secretion [7, 8].

Several points of this proposed mechanism in Figure 9 require attention and analysis, such as the following.

1. The origin and fate of the ouabain-induced vesicles: what is the role of chloride and protons? If a V-ATPase does, indeed, provide the driving force for water accumulation in the vesicles, depletion of intracellular ATP should block both vesicular expansion and ouabain-resistant water extrusion.
2. Published data with liver slices indicated a requirement for ATP derived from oxidative phosphorylation: nevertheless, it is unclear at what extent the decrease of energy charge differently inhibits (a) ouabain-dependent vesicles formation and their transport and secretion to the canalicular pole of the cell, (b) the recovery of intracellular water and ions, and (c) the early recovery of ultrastructure, suggesting a different sensitivity of these functions to the decrease of ATP.

In the next paragraph, we aim to discuss these points.

## 4. Formation of Vesicles: Role of Anions, Protons, and Aquaporins

**Figure 3:**
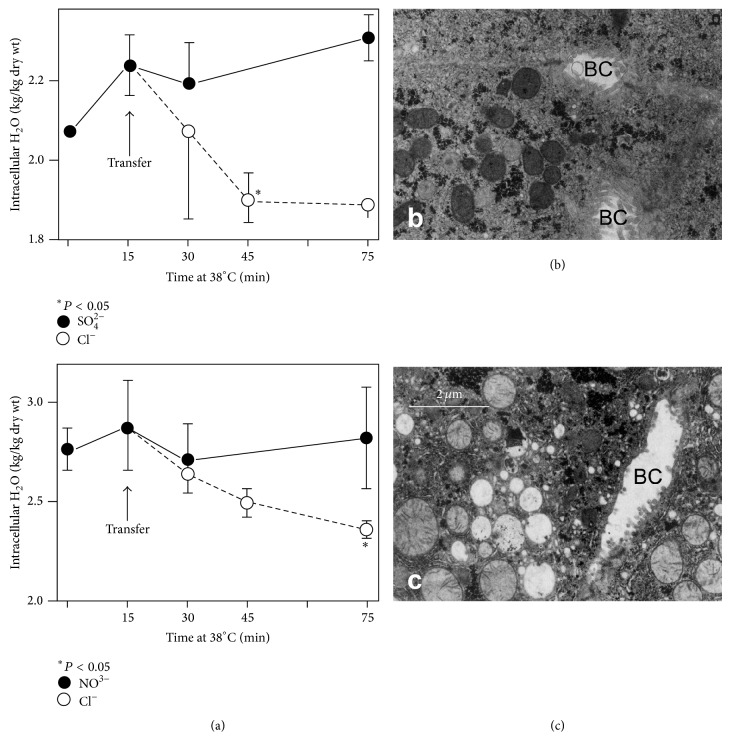
Liver slices were incubated at 1°C for 90 min and then at 38°C for 15 min in a Cl^−^ free medium (SO_4_^2−^ or NO_3_^3−^) and, finally, were transferred in a Cl^−^ containing medium at 38°C for 60 min. (a) Water transport in the presence of ouabain was efficiently prevented by replacement of Cl^−^ in the medium with SO_4_^2−^ (upper panel) or with NO_3_^3−^ (lower panel). (b) At the end of incubation in Cl^−^ free medium in the presence of ouabain, the almost complete absence of typical vesicles was evident. (c) Transferring slices from Cl^−^ free medium to normal medium produces the appearance of ouabain-induced vesicles. BC = bile canaliculus.

**Figure 4:**
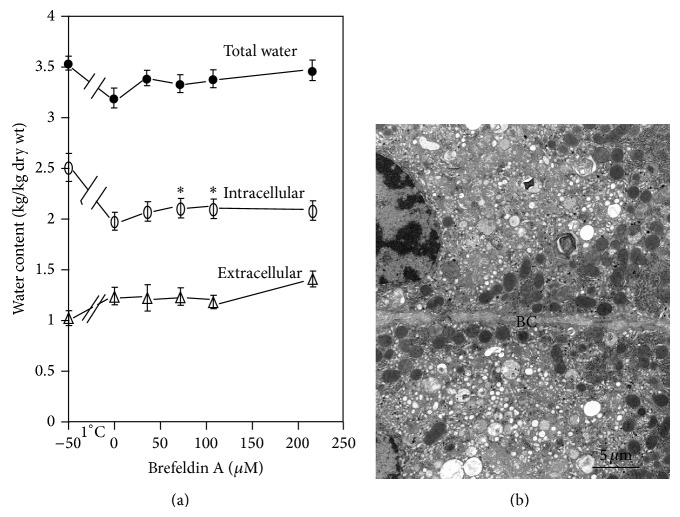
Effects of Brefeldin A on water transport and ultrastructure in the presence of 2 mM ouabain. Liver slices were incubated as described before (see also [7]). (a) Water transport inhibition. (b) Detail of Golgi region of two adjacent hepatocytes: evident Golgi disruption, with only small vesicles left. BC = bile canaliculus.

In the model proposed, in Figure 9, water that accumulates when Na^+^/K^+^-pump is inhibited by ouabain must be delocalized from cytosol and other compartments leading to the formation of vesicles. During a time-course of the recovery at warm incubation, in the presence or absence of ouabain, vesicles appear to originate by expansion of terminal cisternae of the endoplasmic reticulum and of Golgi elements [6] and have acidic contents [32] and their formation is dependent on the presence of Cl^−^ (Figures 3(a) and 3(b)) and vacuolar H^+^-ATPase [33, 34] and, finally, on the integrity of Golgi apparatus, as demonstrated by treatment with Brefeldin A, which prevents vesicles formation (Figures 4(a) and 4(b)), extrusion of water, and recovery of ions [7]. The entry of chloride into vesicles represents the driving force for transport of Na^+^ and water. In fact, replacement of Cl^−^ in the medium with NO^3−^ or SO_4_^2−^ efficiently prevented water extrusion in the presence of ouabain (Table 2) and vesicles were almost absent (Figure 3(b)). Transfer of these slices in a medium containing chloride after only 15 min at 38°C leads to water extrusion (Figure 3(a)) and the appearance of vesicles [24, 25, 30]. The diuretic furosemide, which inhibits cotransport of Na^+^ and Cl^−^, caused effects similar to the absence of chloride [30].

Brefeldin A disrupts the structure and the function of Golgi apparatus and interferes with cell membrane traffic [35, 36]. While Brefeldin A alone had no effects on water and ion content and transport, in the presence of ouabain, 36 *μ*M Brefeldin A partially prevented intracellular water extrusion (Figure 4(a)) and reduced the typical round electron-clear ouabain-dependent vesicles (Figure 4(b)) [7].

Vacuolar proton-dependent ATPase is present in a number of vesicles derived from endoplasmic reticulum and Golgi apparatus [37]. Schisselbauer and van Rossum [33] and van Rossum et al. [8] have explored the transport of protons into vesicles suggesting that this could provide the energy-dependent driving force for chloride transfer from cytosol into the vesicles. Isolated Golgi-derived vesicles accumulate chloride by a mechanism that requires ATP and can be saturated by external (or cytosolic) Cl^−^ [33]. The accumulation is prevented by the anion ionophore tributyltin, suggesting that the uptake of chloride occurs across the membrane against an electrochemical gradient. Its dependence on proton movements is demonstrated by the effect of DCCD (N,N′-dicyclohexylcarbodiimide), a proton ATPase inhibitor, and by the effects of CCCP (carbonyl cyanide m-chlorophenylhydrazone), a specific proton ionophore [34]. In conclusion, protons and a subsequent exchange of H^+^ for cytosolic Na^+^ or K^+^ result in a net uptake of NaCl or KCl which induces water accumulation, confirming that the movements of protons against gradient and the chloride conductance represent the driving force for water movement into the vesicles.

A final question regards the water movements from cytosol (and nucleoplasm) into the vesicles. In the last few years, increasing evidences have demonstrated that water channels aquaporins (AQPs) are the effectors of transmembrane water movements in different mammalian tissues, including liver, kidney, lungs, and various epithelia [38]. The family of AQPs includes 13 members plus a number of isoforms, all involved in water transport and, occasionally, in glycerol transport. Water movements may be transcellular, paracellular, or intracellular:* transcellular* transport occurs at the level of basal or apical pole of a cell to extrude or reabsorb water,* paracellular* transport or exchange occurs at lateral surface of cells through junctional complexes, especially tight junctions, and* intracellular* water movements are responsible for water distribution among various subcellular compartments (i.e., from cytosol and nucleoplasm to various cisternae (endoplasmic reticulum, Golgi apparatus, lysosomes, phagosomes, peroxisomes) or to mitochondrial matrix), implying the presence of APQs in each subcompartment of cell membranes involved in water transport. This occurs in response of osmotic changes and is controlled by AQPs, through posttranslational modifications such as phosphorylation by a AMP/protein kinase which is effective in AQP8 activation and in its trafficking between membranes [39].

**Figure 5:**
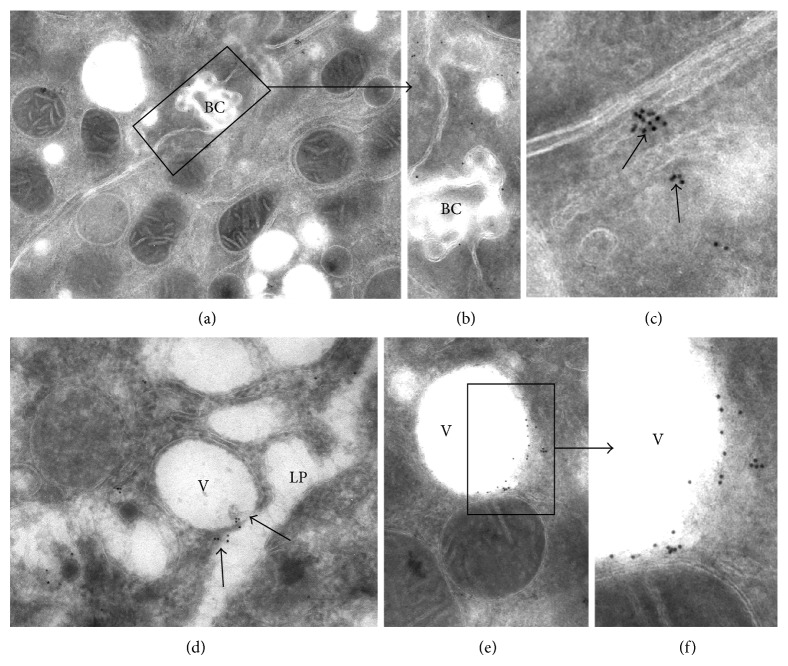
Aquaporin-8 localization in ouabain-induced vesicles and plasma membrane. Liver slices were incubated at 1°C for 90 min and then for 70 min at 38°C in the absence ((a), (b), and (c)) or presence ((d), (e), and (f)) of 2 mM ouabain. At the end of treatments, samples were slightly fixed (5 min in 4% paraformaldehyde) and cryofixed in liquid nitrogen. Ultrathin cryosections were obtained at the cryoultramicrotome, treated with a monoclonal antibody for AQ8 and then with protein A conjugated with 20 nm gold particles. Sections were postfixed and contrasted with osmium tetroxide vapors. (a) Control warm incubation. No gold particles are visible on the vesicle membranes. (b) Control warm incubation. Detail of (a). Few particles are visible on bile canalicular (BC) plasma membrane. (c) Control warm incubation. Occasional clusters of gold particles on the lateral plasma membrane. (d) Control warm incubation in the presence of 2 mM ouabain. A cluster of gold particles is present on the membranes where a typical electron-clear vesicle (V) is fusing with lateral plasma membrane (LP) (arrow). (e) Control warm incubation in the presence of 2 mM ouabain. A number of gold particles are visible on the membrane of an electron-clear vesicle. (f) Control warm incubation in the presence of 2 mM ouabain. Detail of (e) showing the close association between gold particles and vesicular membrane.

Three AQPs have been described in the liver: AQP1 in cholangiocytes, AQP8 in hepatocytes, and AQP9 confined to the sinusoidal (basal pole) membrane of hepatocytes [38]. However, this is a static conception for AQPs. In fact, it is clear that they can rapidly translocate in other compartments in response to osmotic or hormonal stimuli. AQP8 has a role in water movements in/out hepatocyte and among subcellular compartments and in bile formation, before secretion into the bile canaliculus (apical pole of the hepatocyte). Several treatments aimed to increase cAMP (forskolin) rapidly induced redistribution of AQP8 to the plasma membrane, augmenting water permeability [38]. García et al. [39] observed that colchicine, a microtubular inhibitor, blocked the effects of cAMP-dependent translocation, indicating that intracellular AQP8 traffic is microtubule dependent. We have detected AQP8 in the cytoplasm and intracellular vesicles of rat hepatocytes in liver slices by immunoelectron microscopy (Figure 5) (Russo, unpublished results).

In our model of rat liver slices, AQP8, at the end of 90 min cold + 70 min warm incubation in the absence of ouabain, was present in membranes of bile canaliculus (Figures 5(a) and 5(b)) and, in small clusters, into the cytosol or associated with membranes (Figure 5(c)). In the presence of 2 mM ouabain, AQ8 was mostly associated with the typical ouabain-dependent vesicles (Figures 5(e) and 5(f)), even when these vesicles were fusing with plasma membrane (Figure 5(d), arrow), or in cytosolic small clusters (Figure 5(f), arrow). This suggests that the inhibition of Na/K-pump and the increase in intracellular water also stimulate, through an unknown pathway, the redistribution of AQ8. It is also unknown if a de novo synthesis of AQPs may occur.

The growth and enlargement of vesicles in the presence of ouabain is partially dependent on time of incubation and on concentration of ouabain (0.5 to 5.0 mM, reaching the maximal effect at 2 mM) [6].

## 5. Role of Exocytosis: Transport of Vesicles to the Secretory Pole

Ouabain-dependent vesicles, once formed, must be transported and secreted at the apical pole of the hepatocyte, that is, at the bile canaliculus (Figure 9). This requires an intact cytoskeletal system to generate force for polarized transport and a docking system between vesicle membrane and F-actin filaments. In addition, this system should be Ca dependent and ATP consuming, suggesting that the mechanism could be sensitive to the absence of Ca^2+^ and to the decrease of energy charge.

In fact, this step is dependent on the activity of actin components of the cytoskeleton [8]. Russo et al. [7] further explored this aspect for the first time giving evidence that a cytoskeleton-dependent component can contribute to volume regulation even when the Na-K ATPase is inactive.

Cytoskeleton involved in the hepatocyte exocytosis includes two main structures, microtubules [40] and actin microfilaments [41]. In a previous work, the effects of colchicine and other antimicrotubular agents on the ouabain-resistant cell volume control have been explored [24]. At concentrations normally used to disorganize microtubules (1 mM), colchicine was apparently ineffective, while showing some inhibition of vesicle-dependent mechanism when higher concentrations (10 mM) were used. However, these latter concentrations depress ATP synthesis [42] and appeared to be quite toxic as evidenced by large areas of necrosis in particular in the presence of ouabain [24].

The role of actin filaments have been explored by using phalloidin and cytochalasins A, B, D, and E which inhibit actin-dependent processes by, respectively, stabilizing actin filaments (phalloidin) or preventing their elongation (cytochalasins), in both cases resulting in a malfunctioning contractile force generating system.

Each of these agents reduced the extrusion of water, Na^+^, and Cl^−^ in the presence and, to a lesser extent, in the absence of ouabain without affecting the reaccumulation of K^+^ (Table 2). Furthermore, these agents alone also induced the type of vesiculation characteristic of the presence of ouabain. These findings provide a strong evidence in favor of a volume-regulating mechanism which is independent of the Na/K ATPase and also suggest that the vesicular mechanism, in the presence of ouabain, is potentially active also in its absence.

**Figure 6:**
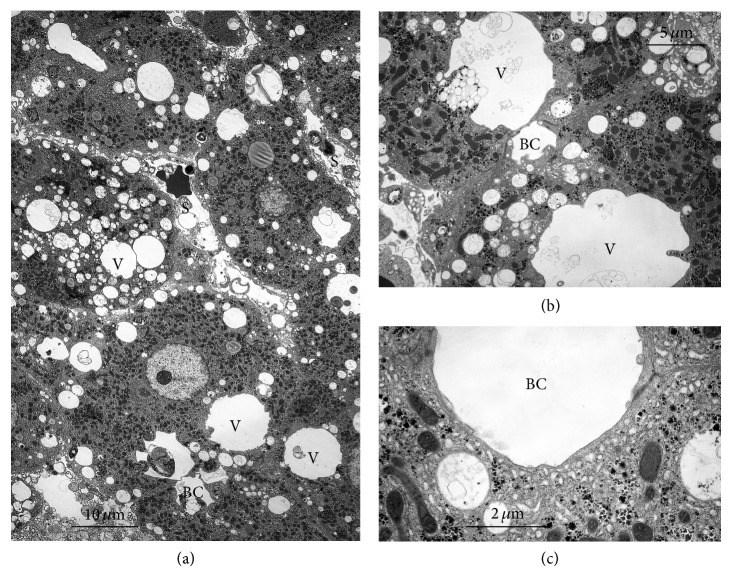
Liver slice incubated as indicated before in the presence of ouabain and cytochalasin D [7]. (a) Numerous vesicles of differing sizes are widely scattered throughout the cells and not particularly oriented to the canalicular region. The large vesicles tend to be more irregular in shape than the small ones probably due to their formation by fusion, and both have rather clear contents. The bile canaliculi appear partially disorganized showing a thin submembranous layer of cortical actin and a loss of microvilli. The perimeter of the canaliculi is round with a smooth surface. (b) Detail of large vesicles. Two cells form an irregular bile canaliculus (BC). Large cytoplasmic vesicles appear to fuse with each other and with the canaliculus. (c) Detail of a bile canaliculus almost devoid of microvilli. The layer of cortical actin is almost unrecognizable because of its thinness. BC = bile canaliculus; V = vesicles; S = sinusoid.

**Figure 7:**
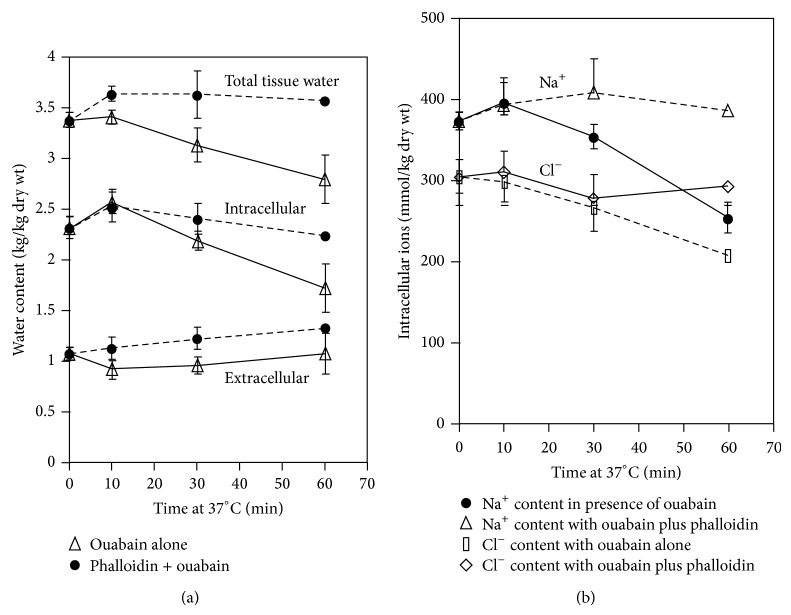
Effect of phalloidin (12 *μ*M) in the presence of ouabain (2 mM) on water (a) and ion (b) content of liver slices incubated for 90 min at 1°C followed by 60 min at 37°C. Water content was paralleled to the retention of Na^+^ and Cl^−^ [7].

**Figure 8:**
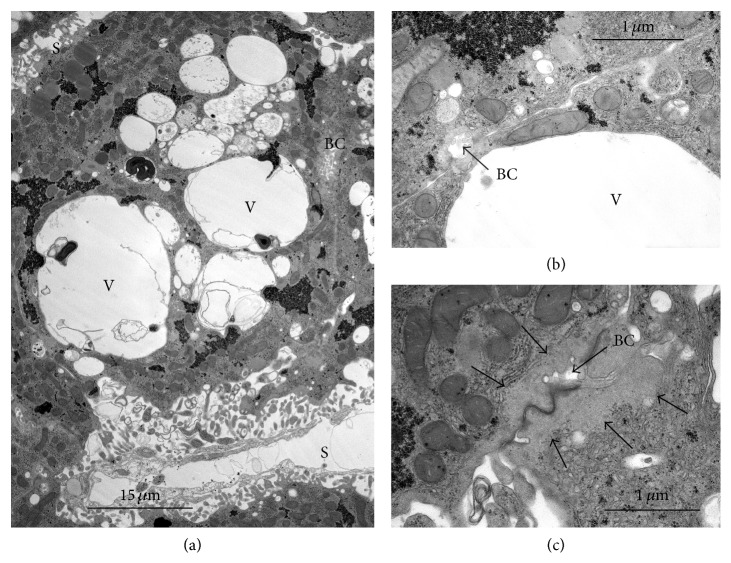
Liver slices were incubated for 90 min at 1°C followed by 60 min at 38°C, with 10 *μ*M phalloidin plus 2 mM ouabain [7]. (a) This micrograph shows an increase in the size and number of vesicles. These show a rather irregular outline probably resulting from the fusion of smaller vesicles. The vesicular contents are clear and similar to that seen with ouabain alone. (b) Detail of a very large vesicle close to a small bile canaliculus. (c) Detail of homogeneous submembrane zone typical of the stabilization effect of phalloidin on cortical actin. BC = bile canaliculus; V = vesicles; S = sinusoid.

**Figure 9:**
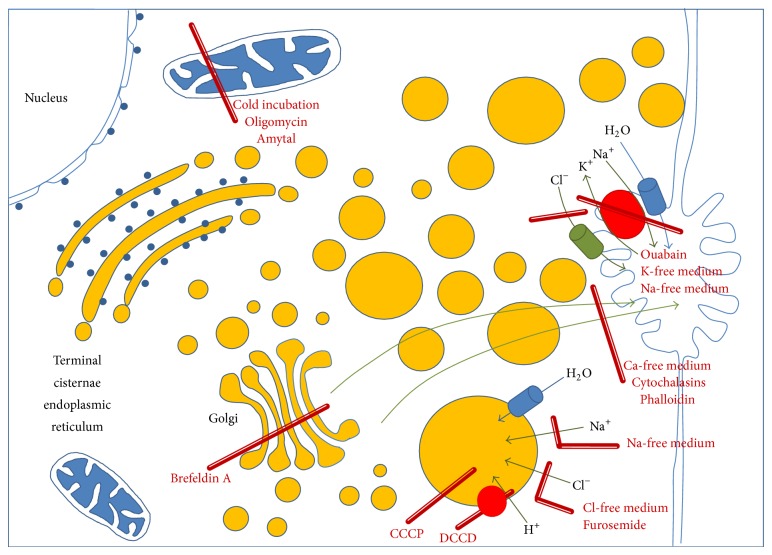
Schematic representation of the ouabain-resistant mechanism of cell volume control, the origin of vesicles, their secretion, and the effects of metabolic inhibitors. CCCP = carbonyl cyanide m-chlorophenylhydrazone; DCCD = N,N′-dicyclohexylcarbodiimide.

In the presence of ouabain, results with phalloidin and cytochalasins A, D, and E were all rather similar to the observations previously obtained with cytochalasin B alone [24]. All these agents inhibited the ouabain-resistant extrusion of total tissue water to varying degrees and caused an increase in the number, size, and area of distribution beyond that seen with ouabain alone (Figures 6, 7, and 8) [7]. Furthermore, the vesicles typically lacked the orientation towards the Golgi and excretory pole as seen with ouabain alone (Figures 6(a) and 8(a)). This distribution suggests that the vesicles are formed but are unable to move on their normal path and to extrude their content at the canalicular pole. The reduced extrusion of total and intracellular water was, in each case, associated with an approximately equimolar reduction of the extrusions of Na^+^ and Cl^−^.

Incubation in the Ca^2+^-free medium caused a partial inhibition of volume recovery (i.e., extrusion of water). However, complete absence of recovery in Ca^2+^-free medium was observed in the presence of ouabain. The reduction of water extrusion seen in the presence of ouabain or absence of Ca^2+^ was due to mainly a slower initial loss. However, when the Ca^2+^-free medium contained ouabain, there was total inhibition which was evident during the first 10 min at 37°C [7]. Thus, these two conditions appear to have an additive effect on the mechanism of water extrusion. Slices incubated in the Ca^2+^-free medium in the absence or presence of ouabain showed a large number of rounded vesicles which were very similar to those seen with ouabain alone, except that they were now more widely distributed throughout the cell without a polarized (Golgi to canaliculus) distribution [7]. At higher magnification, the shape and content of the vesicles and the appearance of bile canaliculi were all very similar with and without Ca^2+^ despite the reduced water extrusion in its absence (Table 2). These observations suggest that (1) Ca^2+^ is not required for the increased formation and expansion of the ouabain-induced vesicles and (2) Ca^2+^ is needed for the vesicles to follow their normal path of distribution in the Golgi and canalicular region and/or to undergo exocytosis, likely regulating the actin/myosin interaction in the molecular motor moving vesicles [43].

The exocytosis of vesicular content in the presence of ouabain has been less convincingly demonstrated in liver than in slices of renal cortex, with few “Ω-shaped” vesicles of fusion between vesicular and plasma. Electron microscopy of liver slices showed that, in the presence of ouabain, the electron-dense markers, OsO_4_ and ferritin, were taken up by basolateral endosomes that fused with the rounded vesicles characteristic of the presence of ouabain. In such slices, electron-dense particles were also present in the bile canaliculi, suggesting exocytotic expulsion of the vesicular contents [24]. In subsequent experiments on fluid-phase endo- and exocytosis, with Lucifer yellow as marker, ouabain somewhat delayed release of the dye. This was consistent with the fusion of Lucifer yellow-containing endosomes with the ouabain-induced vesicles derived from intracytoplasmic membranes, thereby diluting the dye in intravesicular water [31]. It remains to be explored what molecules are specifically involved in docking vesicles to plasma membrane, in the process of fusion, and in opening to the extracellular space.

## 6. Ouabain-Resistant Volume Regulation in Various Mammalian Tissues

This mechanism has been explored in a number of other cell and tissue models with results that are consistent with our proposal. Results have been already published and/or reviewed before [8] in isolated hepatocytes [31], in hepatoma 3924A, a rat liver tumor of the Morris series which retain many morphological and differentiative features of the rat liver [9], kidney cortex [18], in myometrium [15], in lung [32, 44], and in avian salt gland [8]. Interestingly, in all these different models, results were in agreement with our proposed mechanism.

## 7. Corollaries in Human Diseases

The mechanisms of cell volume control and in particular the homeostasis of water are central in mammalian physiology and their alterations are crucial in many human pathological conditions.

Cloudy swelling was recognized by Virchow as cell volume alteration following ischemia, hypoxia, and a number of toxic biological (bacterial toxins) and chemical agents and he clearly stated that extreme cloudy swelling preceded cell necrosis.

Water homeostasis is impaired in edema formation, when intracellular water progressively accumulates in extracellular space of diseased (i.e., inflamed) tissues for vessel permeability alterations.

Hypothermia may cause metabolic inhibition by cold with serious and irreversible damages especially in more susceptible tissues [45]. The lesson learned on water and ion movements and its recovery in the presence of warm exposure may be useful to determine the therapeutic conduct and treatment in case of freezing.

Organ storage for transplant uses hypothermic perfusion to preserve structure and function of organ tissues. Today, it is known that reperfusion at warm is the major responsible reason for cell damage leading to necrosis. Necrotic cells release alarmins which may activate a robust inflammatory response (hyperacute rejection) or trigger the mechanism to activate the chronic rejection. Additionally, the half-life of the transplanted organ, which, for example, in the case of the kidney is about 15 years, can be drastically shortened by the amount of necrosis during recovery from cold perfusion, reducing the number of functionally active cells and increasing the tissue fibrosis.

In conclusion, a better understanding of the mechanisms preserving water and ion homeostasis at cellular level could suggest new molecular targets and strategies to control cell volume, ion composition and structure of cell, and subcellular compartments, modifying the outcome of a number of important pathological conditions or medical situations.

## 8. Conclusions: What Remains to Do?

Vesicular transport of water and ion transport to control cell volume is a multistep mechanism that has been initially hypothesized on the ultrastructural (morphological) basis and then described in detail analyzing and correlating ion composition and ultrastructure in different experimental conditions affecting the various steps of the sequence.

Despite the fact that an amount of data coherent with this hypothesis has been accumulated, there is still a need of direct evidences to address several important points.

1. The* ion composition of vesicles* should be explored by TEM microanalysis (combining X-ray microanalysis and EELA (electron energy loss analysis) spectrometry) on cryoultrathin sections of high pressure cryofixed samples.
2. The* role of aquaporins* in the formation and growth of vesicles needs to be clarified. Different aquaporin isoforms can be constitutively present or rapidly induced in different subcellular compartments allowing an optimal transport and traffic of water to avoid cytosolic and mitochondrial swelling. We still do not know which aquaporin isoforms are involved in different tissues and if they are synthesized* de novo* and/or are translocated from one compartment to another resulting in water redistribution and vesicle formation.
3. The precise mechanism of transport of* ouabain-associated vesicles* to the canalicular pole should be investigated. It seems likely that, considering the central role of actin filaments, a molecular motor F-actin + myosin (myosin V or I) could be the best candidate for vesicles translocation [43]. Additionally, the cargo protein (possibly belonging to* rab* family) attaching vesicle to the myosin should be identified.
4. Several types of* cell stress in human pathology* may show transient or persistent increase of cellular water and changes in ion distribution. It could be extremely interesting for clinical medicine and for better describing this mechanism to understand how cells adapt their gene expression to control this homeostatic mechanism and what are the master transcription factors involved in this adaptation.

## Figures and Tables

**Table 1:** Time-course of (a) water and (b) ionic content of rat liver slices during incubation at 38°C, in the presence or absence of ouabain [6], after 90 min incubation at 1°C. Points at zero time represent the tissue composition at the end of cold incubation at 1°C [8].

Treatment	Time at 38°C (min)	Water (kg/kg dry weight)			Ions (mmol/kg dry weight)		
		Total	Intracellular	Extracellular	Na^+^	Cl^−^	K^+^
Control	0	3.5 ± 0.1	2.7 ± 0.2	1.0 ± 0.1	350 ± 30	320 ± 20	75 ± 10
	15	2.7 ± 0.2	1.6 ± 0.3	1.2 ± 0.1	180 ± 20	175 ± 35	90 ± 12
	60	2.7 ± 0.2	1.5 ± 0.2	1.3 ± 0.1	100 ± 15	180 ± 25	150 ± 15

Ouabain 2 mM	0	3.4 ± 0.2	2.7 ± 0.2	0.9 ± 0.1	360 ± 25	335 ± 20	70 + 10
	15	3.1 ± 0.5	2.2 ± 0.2	1.0 ± 0.2	375 ± 30	350 ± 15	62 + 15
	60	3.1 ± 0.6	2.2 ± 0.4	1.0 ± 0.2	420 ± 10	380 ± 30	50 + 10

**Table 2:** Effect of various treatments on intracellular water content of liver slices in the presence and absence of ouabain.

Intracellular water content (kg water/kg dry wt)^a^						
Incubation^b^						
Treatment	90 min at 1°C	Then, at 38°C for 60 min				
		Control	Treatment	Ouabain	Ouabain plus treatment	(*n*)

Cytochalasin B (100 *μ*g/mL)	2.42 ± 0.33	1.48 ± 0.10	1.60 ± 0.17	1.72 ± 0.10	2.19 ± 0.11^c^	8
Cytochalasin E (33 *μ*g/mL)	2.77 ± 0.07	1.75 ± 0.13	1.92 ± 0.08	1.96 ± 0.06	2.38 ± 0.13^c,d^	18
Colchicine (1 mM)	2.73 ± 0.10	1.68 ± 0.11	1.61 ± 0.08	1.98 ± 0.17	2.15 ± 0.25	10
Cl^−^ free medium, with NO_3_^−^ as substitute	2.60 ± 0.09	1.52 ± 0.08	1.70 ± 0.08	2.00 ± 0.06	2.47 ± 0.13^c^	15

^a^Intracellular water was determined from total tissue water, measured gravimetrically, and the volume of distribution of inulin [9]. Values are the mean ± standard error of the mean.

^b^Slices were incubated in a medium containing 146 mM Na^+^, 5 mM K^+^, 1 mM Mg^2+^, 1.3 mM Ca^2+^, 161 mM Cl^−^, 2 mM phosphate, 1 mM SO_4_^2−^, and Tris (10 mM pH 7.4). It was gassed with O_2_. Cl-free medium contained NO_3_^−^ instead of Cl^−^. Ouabain was at 2 mM. Cytochalasins were added from stock solutions in dimethyl sulfoxide; final concentration in medium was 0.1% (v/v) DMSO.

^c^Significantly greater than value with ouabain alone, by *t*-test; *P* < 0.01.

^d^Significantly less than value after incubation at 1°C, by *t*-test; *P* < 0.01.
